# Fabrication and Characterization of Silk Fibroin/Curcumin Sustained-Release Film

**DOI:** 10.3390/ma12203340

**Published:** 2019-10-14

**Authors:** Xiaoning Zhang, Zhenyu Chen, Hong Bao, Jianwei Liang, Shui Xu, Guotao Cheng, Yong Zhu

**Affiliations:** State Key Laboratory of Silkworm Genome Biology, College of Biotechnology, Southwest University, Chongqing 400715, China; c618@email.swu.edu.cn (Z.C.); dsbrodus@gmail.com (H.B.); liangjianwei0504@163.com (J.L.); 2008uky@gmail.com (S.X.); XZH239@uky.edu (G.C.)

**Keywords:** silk fibroin, curcumin, sustained-release, antibacterial activity, cytotoxicity

## Abstract

In the present work, a sustained-release film composed of silk fibroin (SF), curcumin (Cur), glutaraldehyde (GA), and glycerol (Gly) was prepared successfully for wound dressings. Features relevant to wound dressings of SF/Gly/GA/Cur film were assessed. Physical and chemical properties of the fabricated materials were also characterized. The results showed that the prepared SF/Gly/GA/Cur film demonstrated a good sustained-release performance, flexibility, and gas permeability. In addition, it was found that the prepared SF/Gly/GA/Cur film possessed the capability to effectively inhibit the growth of bacteria and prevent bacterial penetration with a suitable water vapor transmission rate. Furthermore, the prepared composite film was non-cytotoxic, which makes it an ideal material for wound dressings.

## 1. Introduction

Silk fibroin (SF) is a natural polymer material produced by *Bombyx mori* [1]. It is reported that the secondary structures of silk fibroin include random coil, *α*-helix, and *β*-sheet [2], but predominantly in the *β* conformation [3]. Its overall structure is stabilized by extensive hydrogen bonding between *β* sheets [3]. After degumming, silk fibroin fiber can be dissolved in a highly concentrated salt solution. The regenerated silk fibroin solution could be made into various forms including film, powder, hydrogel, fiber, and porous matrix [4]. Silk fibroin-based materials possess good biocompatibility, biodegradability, low inflammatory response, and the ability to accelerate healing in infected wounds [5]. Therefore, SF-based materials have been widely used in biomedical fields, such as tissue engineering [6,7], drug release systems [8,9], implantable electronics [10,11], etc. [12,13].

Among various forms of SF-based materials, SF film is attractive as it possesses oxygen and water vapor permeability [14] and transparency [15] and is easily prepared at a large scale. SF film is mechanically robust and flexible, so it can be wrapped against non-planar objects [16]. In addition, silk fibroin is a perfect substrate for the proliferation and adhesion of a large variety of cells [17]. Therefore, regenerated SF film has been explored for the development of wound dressings and skin substitutes [17,18]. It can act as an artificial skin substitute, covering wounds until split-thickness grafts are available.

Recovery from skin loss is a continuous process that can be divided into three phases: inflammation, proliferation, and tissue remodeling [19]. Wound dressings can provide care in order to expedite healing. One important objective of using a wound dressing is to prevent bacterial infection, as wound contamination can elongate the inflammatory phase and interfere with further phases of healing [20,21]. To limit wound contamination and infection, antimicrobial agents were applied to wound dressings in many cases [22]. It is known that curcumin exhibits strong anti-oxidant, anti-inflammatory, and anti-infective properties [23,24]. The aim of this work was to prepare curcumin sustained-release film by blending curcumin with silk fibroin using the solution casting method and to explore the feasibility of using such composite film for wound healing. The release behavior, morphology, structure, swelling behavior, water vapor transmission rate, antibacterial activity, and cytotoxicity of the curcumin-loaded SF film were investigated in this work. The results together demonstrate that curcumin-loaded SF film is suitable for wound healing applications. 

## 2. Materials and Methods

### 2.1. Materials

Cocoons of silkworm *Bombyx mori* (a Chinese strain demoted as 872) were provided by College of Biotechnology, Southwest University, China. Sodium carbonate and sodium biphosphate dihydrate were purchased from KeLong Chemical Reagent Co., Ltd. (Chengdu, China). Sodium dihydrogen phosphate was purchased from Fangzheng reagent Co., Ltd. (Tianjin, China). Calcium chloride was purchased from Yuanye Bio-Technology Co., Ltd. (Shanghai, China). Curcumin (Cur) was purchased from BBI Life Sciences (Shanghai, China). Ethanol was purchased from Chuandong Chemical Co., Ltd. (Chongqing, China). Potassium bromide was purchased from Sangon Biotech Co., Ltd. (Shanghai, China). Simulated body fluid (SBF) was purchased from Beijing Leagene Biotechnology Co. Ltd. (Beijing, China). Dulbecco’s modified Eagle’s medium (DMEM/HG), PBS (1×), and Penicillin Streptomycin 100× solution were purchased from Hyclone (Logan, UT, USA). Fetal bovine serum (FBS) was purchased from Tianhang Biotechnology Co., (Zhejiang, China). All chemicals are analytical grade and used without further purification. Ultrapure water (resistance > 18 MΩ·cm^−1^) was used in all experiments.

### 2.2. Preparation of SF/Gly/GA/Cur Sustained-Release Film

Regenerated SF solution was prepared as described previously [25]. Cocoons from *Bombyx mori* were boiled for 1 h in an aqueous solution of 0.02 M Na_2_CO_3_ and rinsed with water to extract sericin. The degummed filament of silk fibroin was then dissolved in a ternary solvent of CaCl_2_:CH_3_CH_2_OH:H_2_O, in a molar ratio of 1:2:8, at 70 °C for 3 h. Once the silk fibroin salt solution was cooled down to room temperature, it was then dialyzed (MWCO 8000, Solarbio, Beijing, China) against ultrapure water for 3 days. The obtained SF solution was stirred homogenously at 60 °C in a water bath and condensed to 4% (*w*/*v*). According to the experimental design, 500 μL of curcumin ethanol solution (5 mg/mL), 8 μL of glutaraldehyde (50% *v*/*v*), and 2 μL of glycerol (Gly) solution (50% *v*/*v*) were mixed with 2ml of 4% SF solution. Here, glutaraldehyde (GA) acted as a cross-linking agent [26], and glycerol served as a humectant [27]. After thoroughly mixing, 2.0 mL of the above mixture was pipetted into a 60 mm Petri dish and dried in an artificial climate chamber (HQH-250, Shanghai Yuejin Medical Instrument, China) at 25 °C with a relative humidity at 65% to prepare the SF/Gly/GA/Cur sustain-released film.

### 2.3. Curcumin Release Studies from SF/Gly/GA/Cur Films

The prepared SF/Gly/GA/Cur films were equilibrated at 25 °C with a relative humidity at 65% in an artificial climate chamber for 24 h. Then, the equilibrated samples were placed into weighing bottles containing 10 mL of release medium with compositions of ethanol/PBS (pH = 7.4) solution (V_ethanol_:V_PBS_ = 20:80 or 60:40). Subsequently, the weighing bottles were placed on a shaker at 37 °C with a rotational speed of 100 r/min. Then, 10 mL of the release solution was taken out at certain time intervals and replaced with 10 mL of corresponding fresh release medium. The UV-Vis absorbance of those solutions at 425 nm was measured. 

The amount of curcumin released was calculated according to the following formula [28]:

(1) $$Cur\;release\;\%=\frac{vC_{n}}{m_{0}}\times 100\%$$

where *v* is the volume of release medium removed at a certain time (10 mL), *C_n_* is the concentration of Cur at the displacement time and can be determined from standard calibration curves (can be found in the supplementary material, Figure S1 and Figure S2), *n* is the displacement time, and *m*_0_ is the total mass of Cur in the composite film (2.0 mg). Each experiment was performed in triplicate.

### 2.4. Water Uptake Study

The maximum water uptake of the SF/Gly/GA/Cur film was evaluated. The film was cut into 2 × 2 cm^2^ and was equilibrated at 25 °C with a relative humidity of 65% for 24 h. The weight of this equilibrated film was recorded as *M*_1_; then, the film was immersed in 20 mL of simulated body fluid (SBF) for 24 h. Subsequently, the excess surface water on the film was removed by a filter paper, and the mass of the SF/Gly/GA/Cur composite film was reweighed using a microbalance, recorded as *M*_2_. Five different samples were analyzed, and the average value was used to calculate the maximum water uptake of the SF/Gly/GA/Cur composite film. The equation for the water uptake (*W*) calculation is as below [29]:

(2) $$W=\frac{M_{2}-M_{1}}{M_{1}}\times 100\%$$

### 2.5. Water Vapor Transmission Rate (WVTR)

The samples were mounted onto the weighing bottles, which contained 10 mL of deionized water at the initial time, with an exposed surface area of 2 × 2 cm^2^. Then, the set-ups were placed in an artificial environmental chamber at 37 °C with a relative humidity at 50%. The weights of the set-ups were recorded every 24 h. In order to calculate the water vapor transmission rate (*WVTR*), the weight loss versus time was linearly fitted first, and the following equation was used:

(3) $$WVTR\;\left(\frac{g}{m^{2}\cdot d}\right)=\frac{\left(W_{i}-W_{t}\right)}{\left(A\times t\right)}$$

where *A* is the exposed surface area of sample (4 cm^2^), *W_t_* is the weight of sample at time point of *t*, and *W_i_* is the weight of sample at the initial time.

### 2.6. Material Characterization

Hydrophilicity of the SF/GA/Gly/Cur films was determined by analyzing the water droplet angle formed between liquid/solid interfaces by dropping 8 μL of deionized water onto the sample surfaces. A homemade experimental setup was used for contact angle measurements [30]. The measurement of each contact angle was made within 10 s after each drop to ensure that the droplet did not soak into the composite film. The contact angle value for each sample was taken as the average of three measurements at three different locations of the sample.

In order to characterize the morphology, the samples were mounted on the stubs with double-sided carbon tapes, then sputter coated with gold by using a magnetron plasma sputtering coater (MTI Corporation, Richmond, CA, USA). The samples were examined using a SU8020 scanning electron microscope (HITACHI, Tokyo, Japan).

The SF films were analyzed for their structure with attenuated total reflection Fourier transform infrared (ATR-FTIR, Thermo Scientific Nicolet iN10, Waltham, MA, USA) in the spectral range of 4000–400 cm^−1^ at 4 cm^−1^ spectral resolution and 32 scans. 

The thermal behaviors of the samples were studied using differential scanning calorimetry (DSC, Beijing HengJiu Scientific Instrument Factory, Beijing, China, heating rate: 10 °C/min) in the range of 30–425 °C with a dry nitrogen gas flow of 50 mL·min^−1^. Samples with masses of about 8 mg were placed in Al_2_O_3_ DSC pans and installed in the DSC cell. An empty Al_2_O_3_ pan was used as a reference. 

### 2.7. Antibacterial Experiment

The kinetic analysis of antibacterial activities of the composite film toward both *Staphylococcus aureus* (*S. aureus*, ATCC 25923) and *Escherichia coli* (*E. coli*, ATCC 25922) was carried out in Luria-Bertani (LB) broth in triplicate. Then, 0.1 g of SF/GA/Gly/Cur composite films was immersed in 10 mL LB broth with the initial concentration of bacteria around 10^7^–10^8^ CFU/mL and was incubated at 37 °C with a rotational speed of 100 rpm. At predetermined intervals (2 h, 4 h, 6 h, 8 h, and 12 h), 200 μL of LB culture were withdrawn, and its optical density (OD) value at 600 nm was recorded using an iMark™ Microplate Absorbance Reader (Bio-rad, Hercules, CA, USA). Bacteria inhibition ratios were calculated with the following equation:

(4) $$\mathrm{Inhibition}\;\mathrm{ratio}\;(\%)=\frac{\left(A_{1}-A_{2}\right)}{A_{1}}\times 100\%$$

where *A*_1_ = *A*_control_ − *A*_0_, and *A*_2_ = *A*_t_ − *A*_t_’, in which *A*_control_ is the OD_600_ value of LB broth with initial concentration of bacteria around 10^7^–10^8^ CFU/mL at a designated time; *A*_0_ is the OD_600_ value of sterilized LB broth; *A*_t_ is the OD_600_ value of bacterial culture incubated with the composite film at a designated time; and *A*_t_’ is the OD_600_ value of sterilized LB broth incubated with the composite film at a designated time. 

### 2.8. Bacterial Penetration Test

Bacterial penetration tests on the composite film were performed using *S. aureus* and *E. coli*. In order to do this, 10 μL of bacterial suspension with a concentration of 10^7^–10^8^ CFU/mL was deposited and spread on a 1.5 × 1.5 cm^2^ SF/Gly/GA/Cur film or a same sized SF film that was placed on the LB agar plate. A 1 mL syringe tip (without needle) was used as a liquid spreading tool to prevent the bacterial suspension from exceeding the area of the SF/Gly/GA/Cur film or the SF film. Later, the agar plates were placed at 37 °C in an incubator for 24 h. Then, the SF/Gly/GA/Cur film or the SF film was removed from the plate, and the agar block underneath was cut out from the plate. Subsequently, the agar block was immersed in 15 mL of LB broth and cultured at 37 °C for 2 h with a rotational speed of 100 rpm. 200 μL of LB culture were withdrawn, and OD_600_ values were recorded using a microplate reader at 30 min intervals.

### 2.9. Cytotoxicity Test

The cytotoxicity assay of SF/Gly/GA/Cur sustained-release film was performed against human embryonic kidney (HEK) 293 cell-lines, a type of epithelial cell, with the 3-(4,5-dimethylthiazol-2-yl)-2,5-diphenyltetrazolium bromide (MTT) method as follows [31]. Each sample with a total weight of 0.2 g was cut into small pieces after sterilization with ultraviolet light irradiance in a laminar flow hood of 40 W/cm^2^ for 30 min. Subsequently, each sample was immersed in 10 mL of sterilized Dulbecco’s modified Eagles medium (DMEM) to equilibrate for 72 h at 37 °C and obtain leaching liquor. HEK 293 cells in 100 μL complete growth medium (90% DMEM with 10% FBS) were seeded in a 96-well plate with a density of 2 × 10^4^ cells/well and were cultured for 24 h at 37 °C in a 5% CO_2_ atmosphere. Then,10 μL of leaching liquor was added into each well as sample groups. The HEK 293 cells cultured with complete growth medium served as control groups, and those with complete growth medium containing 0.64% phenol served as positive groups. After 24 h incubation, 10 μL of MTT solution (5 mg/mL in PBS) was added into each well. After 4 h treatment with MTT, the medium was removed. Finally, 200 μL of DMSO was added into each well. Quantitative detection was performed on a Synergy H1 Hybrid plate reader (BioTek, Winooski, VT, USA) at a wavelength of 490 nm. The cell viability was calculated according to equation:

$$\mathrm{Cell}\;\mathrm{viability}\;\left(\%\right)=\frac{{\mathrm{OD}}_{490}\left(\mathrm{sample}\right)}{{\mathrm{OD}}_{490}\left(\mathrm{control}\right)}\times 100\%$$

where OD_490_ (sample) was obtained from the cells cultured in a complete growth medium with leaching liquor of the sample, and OD_490_ (control) was obtained from the cells cultured in a complete growth medium.

To confirm that the prepared SF/Gly/GA/Cur film did not have an adverse influence on the growth and adhesion of cells, morphology and growth of HEK 293 cells on SF/Gly/GA/Cur film was evaluated. The films were cut into 1 × 1 cm^2^. After sterilization with ultraviolet light irradiance, the samples were placed in a 12-well culture plate, and each sample was washed three times with 1 mL of PBS solution (pH 6.5) and each wash was allowed to sit for 5 min to allow for complete diffusion. After removal of PBS solution, 1 mL of HEK 293 cells suspension cells was sampled in each well to reach a cell density of 1 × 10^4^ cells/cm^2^. The cultures were incubated (SANYO Electric Biomedical Co., Ltd., Osaka, Japan) at 37 °C in 5% CO_2_ for 24, 48, and 72 h, and the cell growth morphology was observed under a XDS-1B inverted microscope (Chongqing Optec Instrument Co., Ltd., Chongqing, China).

### 2.10. Statistical Tests

Reported values are the average and standard deviation of *n* = 3 samples, unless otherwise noted.

## 3. Results and Discussion

### 3.1. Optical Transparency Test

**Figure 1:**
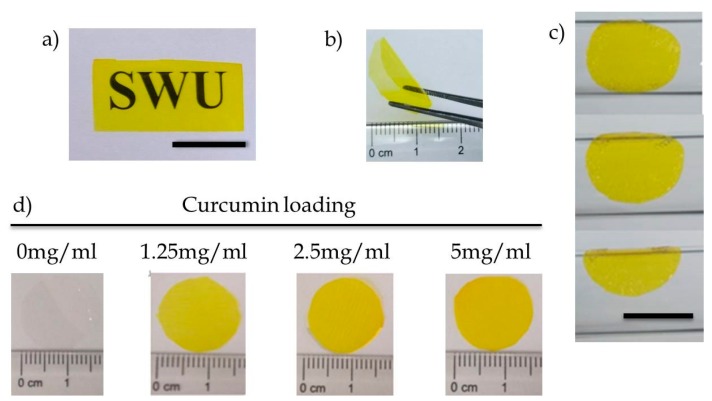
(**a**) Transparency of curcumin-loaded silk fibroin film (the background is white paper printed with the SWU logo, scale bar 10 mm), (**b**) free-standing curcumin-loaded silk fibroin film in the dry state, (**c**) hydrated films follow surface contours (scale bar: 10 mm), and (**d**) silk fibroin films can be loaded with a range of curcumin concentrations.

As Figure 1a demonstrated, the prepared SF/Gly/GA/Cur film allowed good visualization of letters on the print paper, indicating that the composite film was sufficiently transparent for practical use. In addition, dry film was self-standing (Figure 1b), and hydrated films followed surface contours (Figure 1c). Furthermore, by varying the amount of curcumin blended with the silk fibroin solution prior to casting, we could readily control curcumin loading (Figure 1d).

### 3.2. Sustained-Release Performance

**Figure 2:**
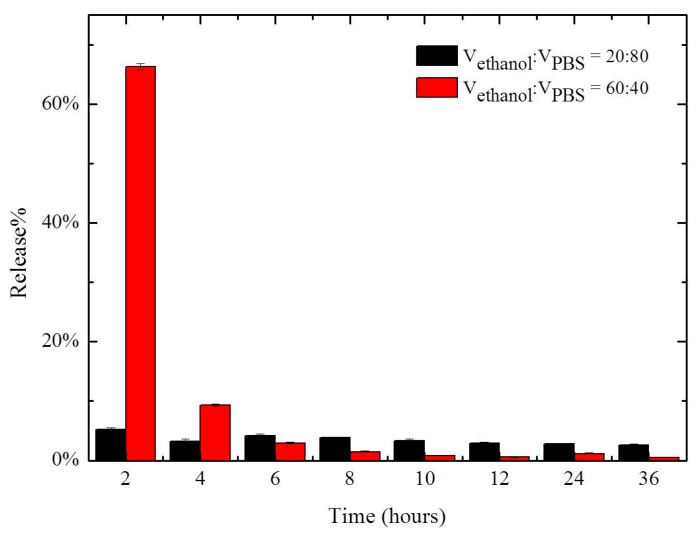
Release of curcumin encapsulated in SF/Gly/GA/Cur composite film versus time.

The release rate of curcumin from SF/Gly/GA/Cur composite film was evaluated in ethanol/PBS solutions. The volume ratios of ethanol and PBS solution used were 20:80 and 60:40. The sustained release performance of SF/Gly/GA/Cur composite film was measured over time and is depicted in Figure 2.

As shown in Figure 2, it was found that curcumin release from the composite film was higher in 60% ethanol/PBS solution compared to that in 20% ethanol/PBS solution at early times. The composite film soaked in 60% ethanol/PBS solution tended to have a rapid initial release. This initial burst lasted for a short time, and then the release of curcumin decreased to a lower stable level. After four hours, the percent release of curcumin from samples soaked in a 60% ethanol/PBS solution was lower compared with that in a 20% ethanol/PBS solution. This can be explained by curcumin’s increased solubility in ethanol compared to PBS. Therefore, inflating the percentage of ethanol in the release medium can result in a “sudden release”. Our observation indicates that the release of curcumin from the composite film can be controlled by the content of ethanol in the release medium.

### 3.3. Water Content Measurements

The water uptake of silk fibroin film is 33.7 ± 1.5%. The SF/Gly/GA/Cur film clearly had a higher affinity for water, with a water uptake of 38.7 ± 1.3%. An increase in water uptake is consistent with the addition of glycerol, which can improve the water-holding capacity of SF film. However, the addition of glutaraldehyde in SF/Gly/GA/Cur film increases the cross-linking density, and the increased crosslinking can reduce swelling ability of the film, in turn limiting the water content.

### 3.4. Water Vapor Transmission Rate (WVTR)

**Figure 3:**
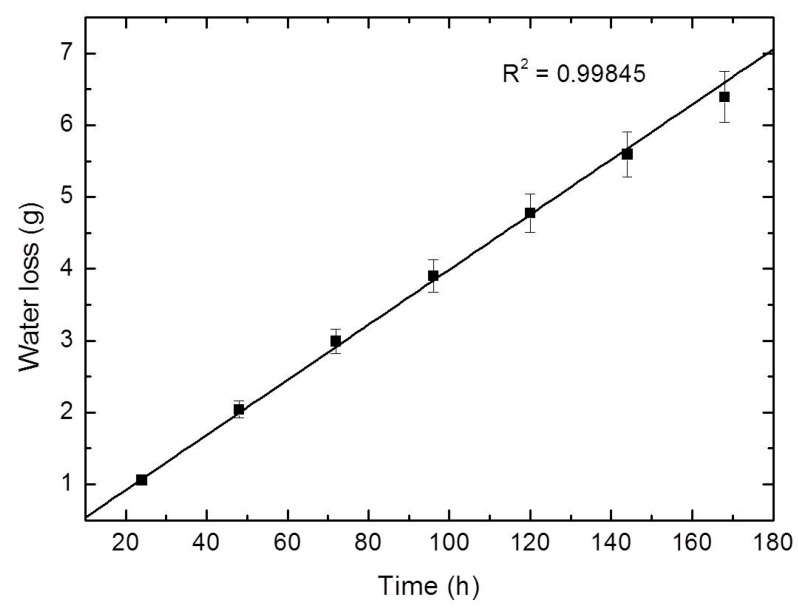
Water vapor transmitted across the SF/GA/Gly/Cur films.

From periodic weighing, a weight loss versus time plot was constructed and is shown in Figure 3. The linearity of the plot confirms the attainment of steady-state operating conditions. The gradient of the plot gives a rate of water transport through SF/GA/Gly/Cur film in gram per hour (g·h^−1^). These values can be used for the WVTR calculation.

SF/Gly/GA/Cur film was found to have a WVTR of 2446.4 ± 72.2 g·m^−2^·day^−1^ which is close to the desired range (2000–2500 g·m^−2^·day^−1^) [32]. Therefore, SF/Gly/GA/Cur film can provide wound areas with suitable moist environment. The clinical problems associated with the dressings having high (dehydration) and low (accumulation) WVTRs may not be experienced with the clinical use of the SF/Gly/GA/Cur film.

### 3.5. Hydrophilicity Measurements

The hydrophilicity of SF/GA/Gly/Cur film was evaluated by water contact angle measurement. The water droplet contact angle on SF/GA/Gly/Cur film surface was measured as 55.0 ± 2.9° indicating that SF/GA/Gly/Cur film surface is hydrophilic. The obtained results imply that SF/GA/Gly/Cur film possesses the capability to serve as a substrate to support cell adhesion, as cells usually attach more readily to hydrophilic surface than to hydrophobic surface [33]. 

### 3.6. Scanning Electron Microscope

**Figure 4:**
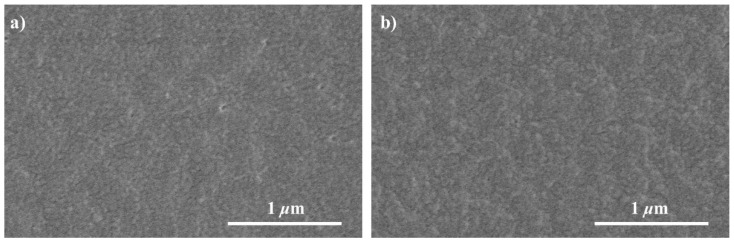
SEM images of the SF film (**a**) and SF/Gly/GA/Cur composite film (**b**).

The morphology of the SF film and SF/Gly/GA/Cur film were investigated by SEM (Figure 4). Both samples formed irregular globule-like structures on their surface. These globule-like structures were formed when the films were prepared with very slow drying [34]. In addition, no heterogeneous structure was observed on the surface of SF/Gly/GA/Cur film, indicating the curcumin powder was fully dissolved and well mixed with SF.

### 3.7. Differential Scanning Calorimetry Analysis

**Figure 5:**
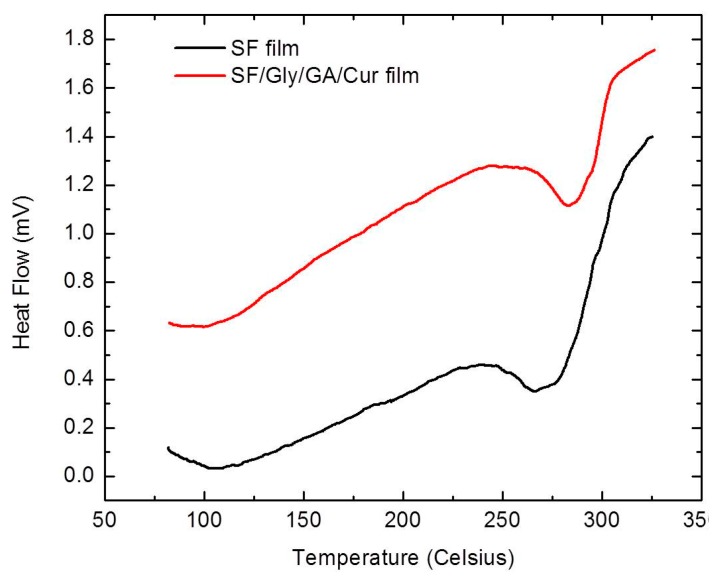
DSC curves of the SF film and SF/Gly/GA/Cur composite film.

The thermal properties of SF films and SF/GA/Gly/Cur composite films were examined through DSC (Figure 5). During the heating scans, all samples first showed a bound water evaporation peak around 100 °C (373.15 K), which can be attributed to the loss of free water trapped inside [35].

In addition, the SF films have clearly shown the degradation peak temperature at 268.1 ± 1.9 °C. The SF/GA/Gly/Cur composite films had a degradation peak around 287.3 ± 2.5 °C, indicating that the SF/GA/Gly/Cur composite films were more thermally stable than the SF films. The improved thermal stability can be attributed to the blending of GA which can lead to a cross-linking of SF, therefore resulting in a greater thermal stability of SF/GA/Gly/Cur films.

### 3.8. Infrared Spectroscopy Analysis

**Figure 6:**
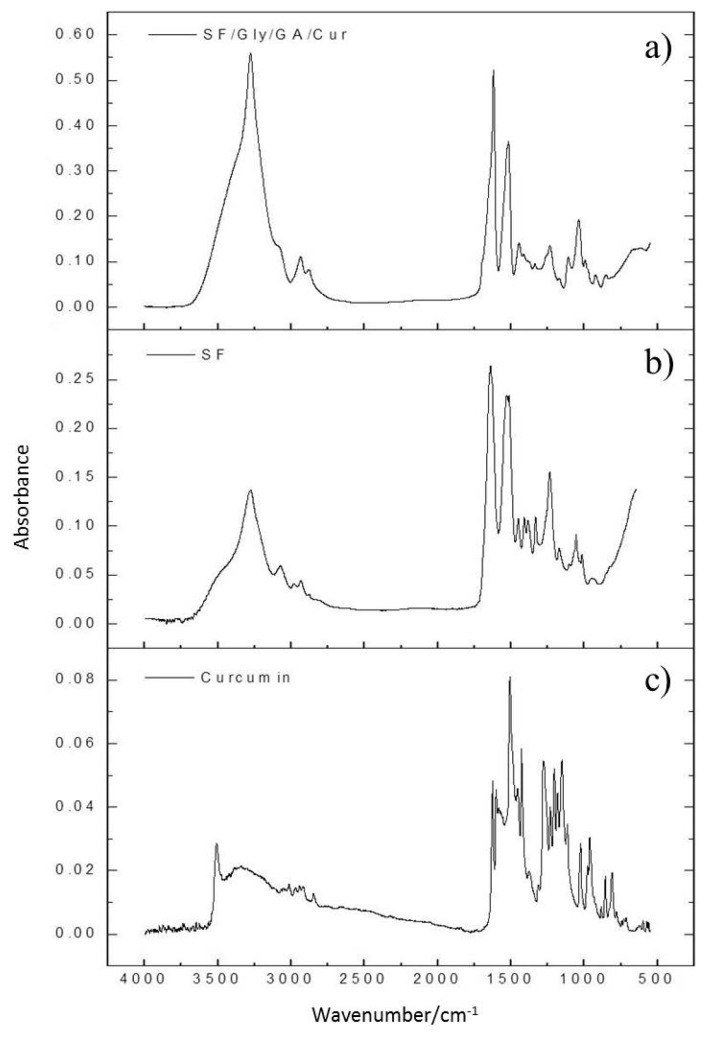
FTIR spectra of the SF/Gly/GA/Cur composite film (**a**), the SF film (**b**), and the pure curcumin (**c**).

The FTIR spectra of SF and SF/Gly/GA/Cur films are demonstrated in Figure 6. Both SF and SF/Gly/GA/Cur films showed signals at 1600–1690 cm^−1^, 1480–1575 cm^−1^, and 1229–1301 cm^−1^ for amide I, II, and III respectively [36]. Spectrum for SF/Gly/GA/Cur film did not show any of the signals that were seen in curcumin spectrum.

**Figure 7:**
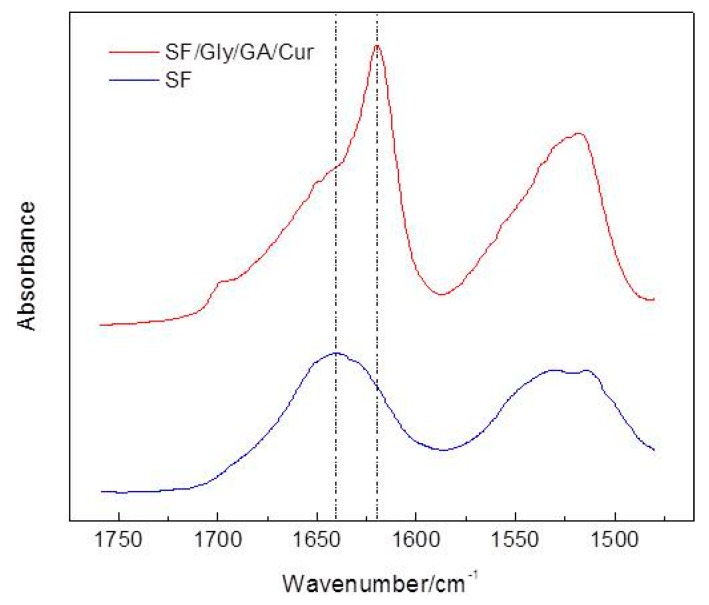
Zoom-in of Figure 6a,b for the region from 1480 cm^−1^ to 1760 cm^−1^.

As can be observed from Figure 7, the band of 1640 cm^−1^ shifted to 1620 cm^−1^ with a shoulder at 1640 cm^−1^ for amide I, after blending SF with curcumin, glutaraldehyde, and glycerol for SF/Gly/GA/Cur film preparation. As the frequency range of 1618–1640 cm^−1^ was assigned to the enriched *β*-sheet structure in silk fibroin [37,38,39], and the band at 1620 cm^−1^ can be attributed to the parallel *β*-sheet conformation [40], our results verified the changes in the secondary structure of SF and revealed the conformation of SF transit from a poorly defined *β*-sheet structure to the well-ordered parallel *β*-sheet conformation after blending with curcumin, glutaraldehyde, and glycerol.

In addition, a sharper and more intense peak at 3273 cm^−1^ after mixing SF with curcumin, glutaraldehyde, and glycerol suggested increased hydroxyl groups [41]. Hydroxyl groups can act as hydrogen-bond donors in hydrogen bond formations. The increased amount of hydroxyl groups could promote the formation of intermolecular hydrogen bonds, endowing stability to the prepared SF/Gly/GA/Cur composite films, which is in accordance with the DSC results that SF/Gly/GA/Cur composite film possesses greater thermal stability. 

### 3.9. Antimicrobial Activity Test

**Figure 8:**
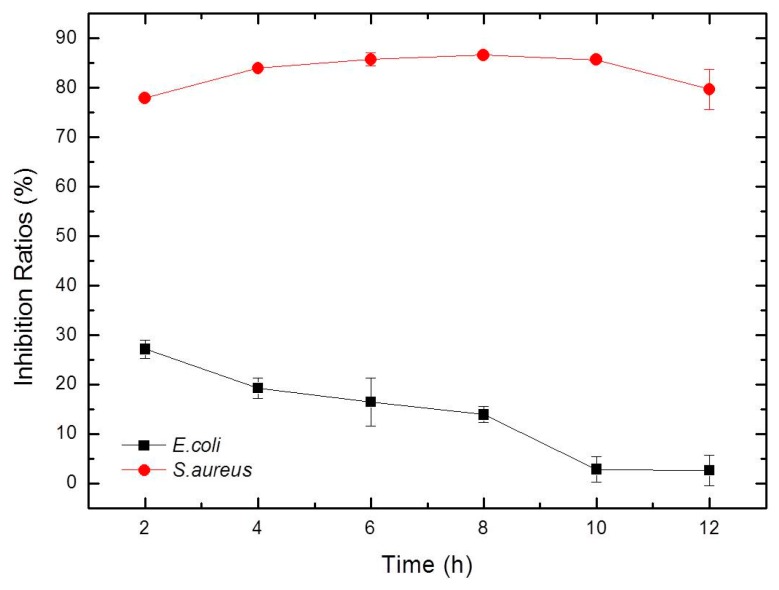
Inhibition ratio kinetic curves of SF/Gly/GA/Cur composite film against *S. aureus* and *E. coli*.

It was reported that SF without antibacterial agent does not exhibit antibacterial activity [42,43]. In this work, Gram-positive bacteria *S. aureus* and Gram-negative bacteria *E. coli*, which are common bacteria found in wound infections [44], were used to test the antimicrobial effectiveness of the SF/GA/Gly/Cur composite film. The bacterial inhibition ratios of the SF/GA/Gly/Cur composite film against *S. aureus* were all higher than 70% during the 12 h of incubation, indicating that the composite film possesses good *S. aureus* inhibition. The inhibition ratios of the composite film against *E. coli* were not as effective as those against *S. aureus* (Figure 8). The differences for SF/Gly/GA/Cur composite film against Gram-positive bacteria *S. aureus* and Gram-negative bacteria *E. coli* could be attributed to the fact that curcumin has favorable antibacterial activity in vitro against *S. aureus*, but unfavorable against *E. coli* [45].

### 3.10. Bacterial Penetration Ability and Barrier Function Test

**Figure 9:**
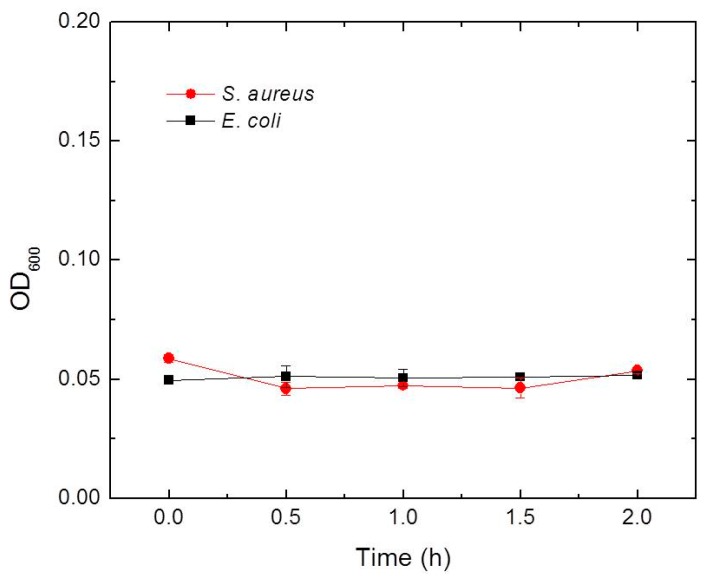
Growth curves of *S. aureus* and *E. coli* from agar blocks under the SF/GA/Gly/Cur composite film incubated in LB broth for 2 h as described in text.

In daily wound care, if the dressing cannot effectively insulate against the invasion of external bacteria, it is easy to cause secondary bacterial infection [46]. Therefore, we analyzed the protection ability of SF/GA/Gly/Cur composite film against secondary bacterial infection. By following the procedure described in the experimental section, the LB cultures were checked at 0 min, 30 min, 60 min, 90 min, and 120 min respectively. The results (Figure 9) showed no bacterial growth (both *S. aureus* and *E. coli*) in the LB culture, which indicates that the SF/GA/Gly/Cur composite film is an effective bacterial barrier. This bacterial barrier feature is attributed to ethanol from the curcumin ethanol solution which can convert SF from a water-soluble state to a water-insoluble structure [47]. The insolubility of the SF/GA/Gly/Cur composite film in water blocks the penetration of bacterial suspension through the SF/GA/Gly/Cur composite film. Our observation also suggested that the SF film cannot protect the area underneath from the penetration of bacteria (can be found in the supplementary material, Figure S3).

### 3.11. Cytotoxicity Test

**Figure 10:**
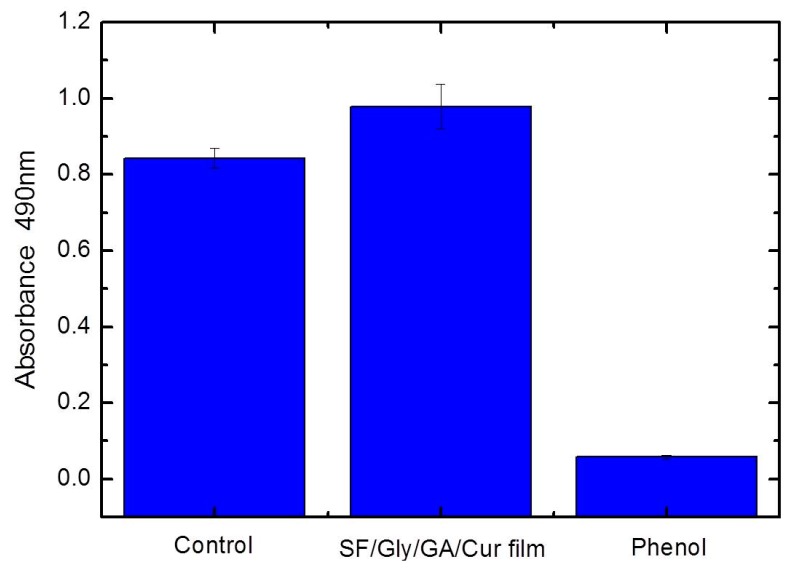
Viability of HEK 293 cells after 24 h of contact with leaching liquor obtained from SF/GA/Gly/Cur composite film, and revealed increased cell viability compared to control. The error bars denote the standard error of the mean (*n* = 3).

Cell viabilities of the SF/GA/Gly/Cur composite film was evaluated and compared. The values were determined using the standard MTT proliferation test at 37 °C for 24 h. The cells were treated with leaching liquor from the SF/GA/Gly/Cur composite film, and the cell viability was measured through MTT absorbance at 490 nm. The relative cell proliferation rate of sample groups compared to that of control groups was 116.2 ± 7.9% (Figure 10). This result revealed that SF/GA/Gly/Cur composite film exhibited good biocompatibility. 

**Figure 11:**
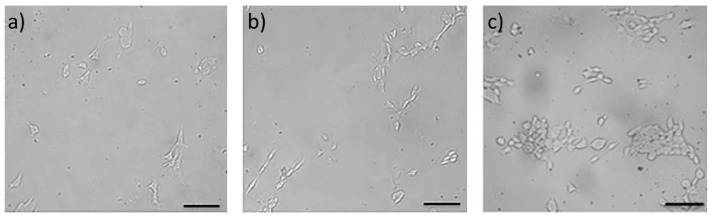
Using an optical microscope to observe morphology of HEK 293 cells cultured on sterilized SF/GA/Gly/Cur composite films with a cell seeding density of 10^4^ cells/cm^2^ after 24 h (**a**), 48 h (**b**), and 72 h (**c**) of culturation. Scale bars: 100 μm.

To test the interaction between cells and the SF/GA/Gly/Cur composite film, the cell adhesion morphology was captured by optical microscope. As can be observed from Figure 11a, cells on the SF/Gly/GA/Cur composite film were randomly distributed 24 h after seeding, and most of the cells spread on the surface of the SF/Gly/GA/Cur composite film were in a different manner. At the second checkpoint (48 h after cell seeding), cells on the SF/Gly/GA/Cur film demonstrated an elongated shape (Figure 11b). In addition, individual cells were in direct contact with other cells at this time. At 72 h after cell seeding, cells on the SF/Gly/GA/Cur film started to form a monolayer, and the number of the cells visibly increased (Figure 11c). The results suggest that the SF/GA/Gly/Cur composite film is biocompatible without rejecting cells, and is able to promote cell growth and colonization, which outperforms the SF film (can be found in the supplementary material, Figure S4). Therefore, the SF/GA/Gly/Cur composite films can be potentially used as wound dressings.

## 4. Conclusions

In this work, a SF/Gly/GA/Cur sustained-release film was successfully prepared by using SF as the substrate, GA as the cross-linking agent, and Gly as the humectant. The water content of the prepared composite film was approximately 39%. The curcumin release rate could be controlled by varying the volume ratio of ethanol in the release medium. The prepared SF/GA/Gly/Cur composite film was characterized by SEM, FTIR, and DSC. The results showed that the surface of the SF/Gly/GA/Cur film was compact, smooth, and had no irregularities, indicating excellent compatibility of components. Additionally, the secondary structure of SF/Gly/GA/Cur film possessed a well-ordered confirmation with higher thermal stability. The prepared SF/Gly/GA/Cur film could provide wound areas with a suitably moist environment, exhibited remarkable antibacterial activity against *S. aureus*, and acted as an effective protective barrier to inhibit the penetration of bacteria. In addition, MTT assay results demonstrated that the prepared SF/GA/Gly/Cur composite film was biocompatible to HEK 293 cells. In conclusion, this work provided a simple method of fabricating a novel SF/Gly/GA/Cur composite film that possesses considerable potential for clinical applications in wound healing.

## Supplementary Materials

The following are available online at https://www.mdpi.com/1996-1944/12/20/3340/s1, Figure S1: The linear calibration curve was acquired by plotting UV-Vis absorbance of 3 mL of 0.25, 1, 1.5, 2, 2.5 μg/mL curcumin in 20% (*v*:*v*) ethanol: PBS solution; Figure S2. The linear calibration curve was acquired by plotting UV-Vis absorbance of 3 mL of 0.25, 1, 1.5, 2, 2.5 μg/mL curcumin in 60% (*v*:*v*) ethanol: PBS solution; Figure S3: Growth curves of *S. aureus* and *E. coli* from agar blocks under the as-prepared SF film incubated in LB broth for 2 h as described in text; Figure S4: Using an optical microscope to observe morphology of HEK 293 cells cultured on sterilized SF films with a cell seeding density of 10^4^ cells/cm^2^ after 72 h of culturation. Scale bars: 100 μm.
