# Supplementary material for: Fabrication and Characterization of Silk Fibroin/Curcumin Sustained-Release Film
Source: Materials (Basel). 2019 Oct 14;12(20):3340. doi: 10.3390/ma12203340 (PMC6829413; doi:10.3390/ma12203340)
Supplement: Supplementary file 1 [file materials-12-03340-s001.pdf]

materials

Supplementary Materials

Fabrication and Characterization of Silk Fibroin/Curcumin Sustained-Release Film

Xiaoning Zhang *, Zhenyu Chen, Hong Bao, Jianwei Liang, Shui Xu, Guotao Cheng and Yong Zhu *

State Key Laboratory of Silkworm Genome Biology, College of Biotechnology, Southwest University, Chongqing 400715, China; c618@email.swu.edu.cn (Z.C.); dsbrodus@gmail.com (H.B.); liangjianwei0504@163.com (J.L.); 2008uky@gmail.com (S.X.); XZH239@uky.edu (G.C.)
* Correspondence: xzhang@swu.edu.cn (X.Z.); xzhan21@ilstu.edu (Y.Z.); Tel.: +86-1592-281-5612

Various concentrations of curcumin in release medium were prepared by successive dilution of a curcumin-release medium solution of known concentration, and the UV-Vis absorbance of above curcumin solution at 425nm [1] were measured by a UV–Vis absorption spectrophotometer (T6, Beijing Puxi Analytic Instrument Ltd., China) in order to build a standard curve for calculation of curcumin released from SF/Gly/GA/Cur films.

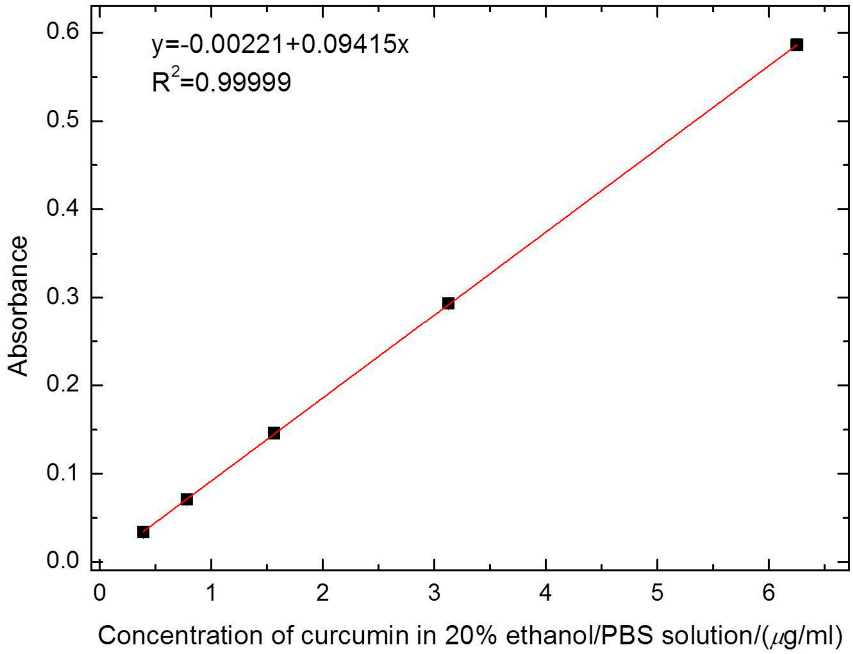

Figure S1. The linear calibration curve was acquired by plotting UV-Vis absorbance of 3 mL of a series of known concentrations of curcumin in 20% ethanol/PBS solution (v/v).

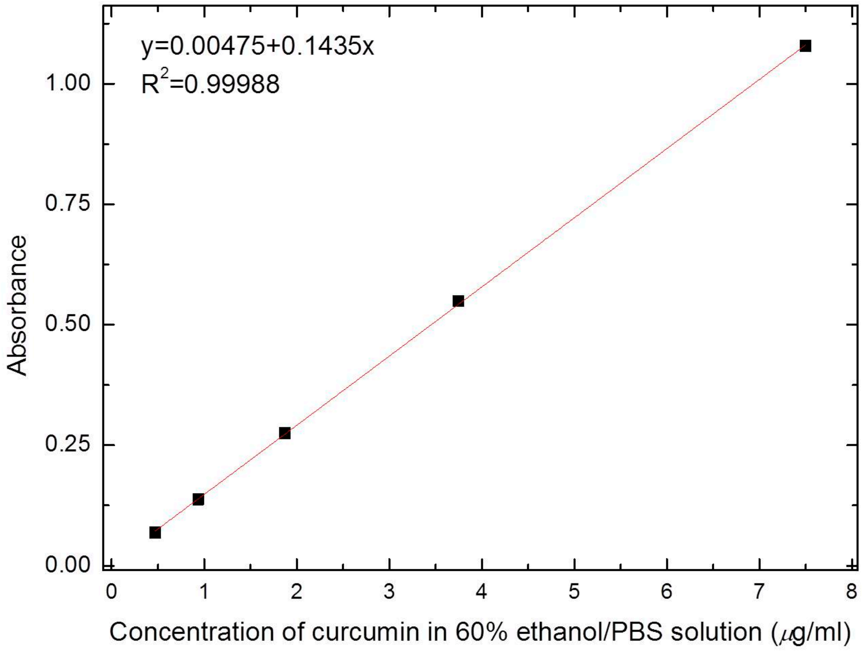

Figure S2. The linear calibration curve was acquired by plotting UV-Vis absorbance of 3 mL of a series of known concentrations of curcumin in 60% ethanol/PBS solution (v/v).

Bacteria penetration experiment was carried out on the SF films. As SF films were soluble in water and easily dissolved once the bacterial suspension was placed on them, they cannot protect the area underneath from the penetration of bacteria.

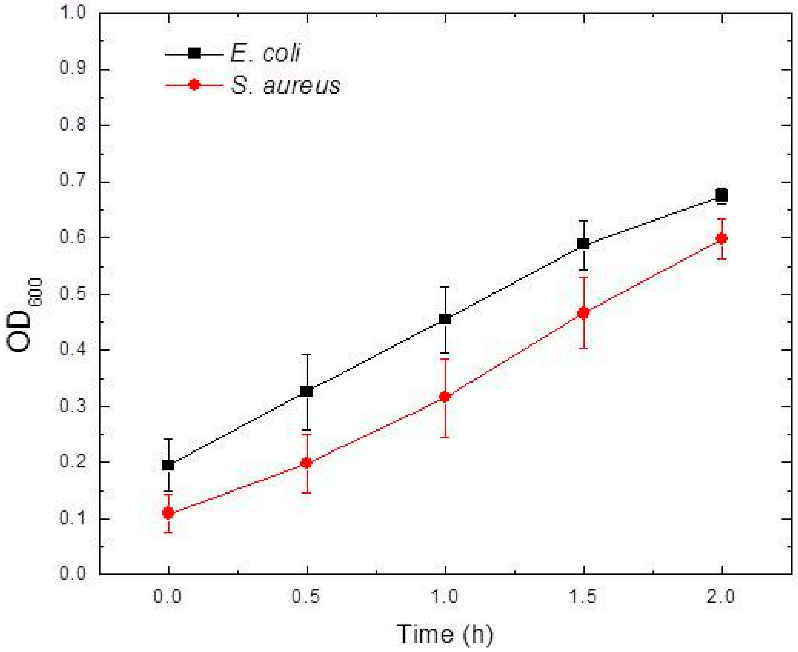

Figure S3. Growth curves of *S. aureus* and *E. coli* from agar blocks under the SF films incubated in LB broth for 2 hours as described in text.

Figure S4 is the optical microscope photograph taken to evaluate cell adhesion on the SF film through cell adhesion morphology. As can be observed, cell adhesion morphology was seem to be mainly rounded on the SF film, and the cells on the SF film stayed assembled as cell clusters, 72 hours after cell seeding.

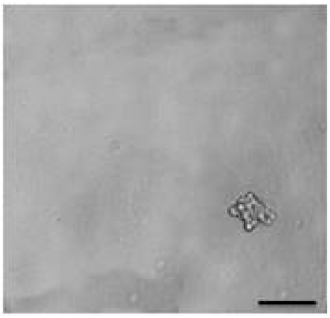

Figure S4. Using an optical microscope to observe morphology of HEK 293 cells cultured on sterilized SF films with a cell seeding density of 10^4 cells/cm^2 after 72 hours of culturation. Scale bars: 100 μm.

Reference

1. Hoda, N.; Naz, H.; Jameel, E.; Shandilya, A.; Dey, S.; Hassan, M.I.; Ahmad, F.; Jayaram, B. Curcumin specifically binds to the human calcium-calmodulin-dependent protein kinase iv: Fluorescence and molecular dynamics simulation studies. *Journal of Biomolecular Structure & Dynamics* **2016**, *34*, 572-584.
